# Navigating work and life– a qualitative exploration of managers’ and employees’ views of return-to-work after sick leave due to common mental disorders

**DOI:** 10.1186/s12889-024-17765-x

**Published:** 2024-02-05

**Authors:** Lisa Holmlund, Helena Tinnerholm Ljungberg, Ute Bültmann, Elisabeth Björk Brämberg

**Affiliations:** 1 Institute of Environmental Medicine, Unit of Intervention and Implementation Research for Worker Health, Karolinska Institutet, SE-171 77 Stockholm, Box 210, Sweden; 2 Department of Neurobiology, Care Sciences and Society, Division of Occupational Therapy, Karolinska Institutet, Fack 23 200, SE-141 83 Stockholm, Huddinge Sweden; 3 Department of Health Sciences, Community and Occupational Medicine, University of Groningen, University Medical Center Groningen, Hanzeplein 1, 9700 RB Groningen, The Netherlands; 4 School of Public Health and Community Medicine, Institute of Medicine, University of Gothenburg, SE-405 30 Gothenburg, Box 100, Sweden

**Keywords:** Common mental disorders, Everyday life, Return-to-work, Work-home interference, Work-family conflict, Work-life balance

## Abstract

**Background:**

Incorporating multiple perspectives and contexts in knowledge mobilisation for return-to-work after sick leave due to common mental disorders can promote interprofessional and organisational strategies for facilitating the return-to-work process. This study aimed to explore the facilitators of and barriers to return-to-work after common mental disorders. This exploration considered the perspectives of employees and managers and the realms of work and private life.

**Methods:**

A qualitative approach was used with data from 27 semi-structured telephone interviews. The strategic sample consisted of employees who returned to work after sick leave due to common mental disorders (*n* = 17) and managers responsible for their return-to-work process (*n* = 10). Thematic analysis conducted in a six-step process was used to generate themes in the interview data.

**Results:**

The analysis generated three main themes with subthemes, illustrating experiences of barriers to and facilitators of return-to-work positioned in the employees’ private and work contexts: (1) Getting along: managing personal difficulties in everyday life; (2) Belonging: experiencing social connectedness and support in work and private life; and (3) Organisational support: fostering a supportive work environment. The results contribute to a comprehensive understanding of the return-to-work process, including the challenges individuals face at work and in private life.

**Conclusions:**

The study suggests that return-to-work after sick leave due to CMDs is a dynamic and ongoing process embedded in social, organisational, and societal environments. The results highlight avenues for an interprofessional approach and organisational learning to support employees and managers, including space for the employee to recover during the workday.

**Trial registration:**

This study recruited employees from a two-armed cluster-randomised controlled trial evaluating a problem-solving intervention for reducing sick leave among employees sick-listed due to common mental disorders (reg. NCT3346395).

## Introduction

Return-to-work (RTW) after sick leave due to common mental disorders (CMDs) such as mild to moderate depression, anxiety, adjustment, and stress-related disorders is essential for an employee’s health, participation in society [1, 2], and for reducing societal costs associated with sick leave [3]. Multiple studies have emphasised the RTW process as a collaborative effort of stakeholders positioned within different systems and environments [4, 5]. The workplace system plays a vital role in employee health [6, 7] because both theory [8, 9] and empirical research [10–12] suggest that high psychosocial job demands are associated with sick leave due to CMDs and a prolonged RTW. In addition, recent research has emphasised the importance of taking a broader perspective that considers everyday life, including work-home interference, to better understand sick leave [13–15] and the RTW process [10, 16]. To ease RTW after a CMD, organisational and managerial elements should be considered in accommodating the employee [6]. Additionally, it is essential to consider the various everyday contexts of work and private life in which the RTW process occurs.

Understanding the worker’s relationship with their environment is relevant for that person’s possibility to return to and maintain work after sick leave [17, 18], and whether relations are favourable or unfavourable can potentially facilitate or hinder the RTW process. The environment in which RTW occurs is often defined according to multilevel paradigms, including personal, social, organisational, and societal levels [17, 18]. Within these levels, actions and interactions between stakeholders are ongoing through phases of the RTW process [4, 19], e.g. the therapeutic phase and the actual RTW. Nevertheless, research has focused on single aspects of the person *or* the environment. For example, low symptom severity, no previous sick leave episodes, positive RTW expectations, attitudes, and high self-efficacy are personal aspects associated with favourable RTW outcomes [20, 21]. Within social levels, good relationships with the manager, co-workers [16, 22, 23], family, and friends [16, 22] are considered essential for successful RTW. However, the evidence for social support at work is still inconclusive [10–12, 24]. On the organisational level, high psychological demands pose a primary barrier to RTW [10–12]. Additionally, insufficient collaboration among actors in the RTW process and bureaucracy pose organisational and societal challenges for RTW [16, 22, 25, 26]. To facilitate RTW, organisational and managerial strategies are deemed critical [18, 27], along with effective communication and coordination among stakeholders [28].

Employer and managerial roles are important, given an employer’s responsibilities for the employee in the RTW process. The employer is responsible for the implementation of policies and routines regarding RTW. For example, in Sweden, an employer is accountable for developing a RTW plan within 30 days of the start of sick leave if the sick leave is anticipated to exceed 60 days. The manager’s role includes being well-informed about policy and procedures, being operative in contacting and communicating with the employee through sick leave and RTW [4, 19], selecting accommodations for the employee, and following the RTW plan [4]. Typically, good communication between the manager and the employee is imperative for the RTW process [19, 23, 27, 29, 30] and is associated with better RTW outcomes [31]. Moreover, the managers’ knowledge of and experience with organisational policies regarding RTW have been identified as supportive factors for managers in the RTW process [27]. However, for good communication and support, managers ascribe responsibility to their employees to take an active role in RTW, communicate and articulate their needs throughout the process, and adhere to the RTW plan [4]. Managers face the delicate task of balancing the various pressures from employees, co-workers, and top management [23, 27, 29]. Despite comprehensive responsibilities and the general willingness of managers to support employees on sick leave due to CMDs [29], studies point to the managers’ lack of training and autonomy for providing support during RTW [23, 27, 29], and managers’ ignorance of employees’ experiences and a tendency to individualise problems rather than address organisational deficiencies [27].

While work is a significant part of most adults’ daily lives, focusing solely on work can limit understanding of the factors affecting an individual’s health [32, 33]. Therefore, moving beyond work and considering other aspects of a person’s life is essential for understanding the RTW process. For instance, qualitative research has shown that the double burden of work and private life demands is a barrier to RTW [16, 34] and that there are gender differences relating to women’s greater responsibilities for domestic and emotional work in their private lives [16]. Additionally, studies have revealed that women found home-related changes, such as increased support at home, important for their recovery [35]. In quantitative research, work-home interference is typically defined as spill-over from one domain to another. Studies have shown that high work-to-home and home-to-work interference levels are associated with an increased risk of sick leave due to burnout and stress-related mental health issues for both men and women [14, 15]. Recently, high levels of work-to-home interference have been linked to prolonged RTW [10, 24].

A more comprehensive view of the RTW process, including work and private life, from the perspective of employees and managers, who are critical stakeholders in the RTW process, has rarely been applied in research. Such perspectives could broaden our understanding of the RTW process and add to research on gender differences in RTW [16]. Exploring RTW from the perspective of managers and employees can further provide insights into the complex RTW process after sick leave due to CMDs and help develop useful interprofessional and organisational strategies for RTW. Therefore, the present study aimed to explore the facilitators of and barriers to RTW after common mental disorders. This exploration considered the perspectives of employees and managers and the realms of work and private life.

## Methods

This study used a qualitative, explorative design and constructivist epistemology. Data included semi-structured interviews with employees and managers and were analysed thematically [36, 37]. The authors constitute an interdisciplinary team of researchers with expertise in qualitative research and research centred on gender, everyday life, sick leave, and RTW. All authors are women and hold positions at universities in Sweden and the Netherlands. The reporting of the study follows the Consolidated Criteria for Reporting Qualitative Studies as recommended by Tong et al. [38].

### Participants and procedure

In total, 27 participants (*n* = 17 employees and *n* = 10 managers) were recruited. Employees were recruited from a randomised controlled trial evaluating the effectiveness of a problem-solving intervention among employees on sick leave due to CMDs (PROSA) [39]. In PROSA, the participant eligibility criteria were age 18–59, 2–12 weeks of sick leave due to mild to moderate depression, anxiety, or adjustment disorder, and acceptance of manager involvement. Exclusion criteria in PROSA were severe depression, other severe mental disorders (e.g., psychotic or bipolar disorders, or referral to a psychiatrist), pregnancy, somatic complaints or disorders that affect workability, or an inability to read, write, and understand Swedish [39]. In the present study, an additional eligibility criterion for employees applied: employees should, at some point between inclusion and the interview, have returned to paid work or have been at on-the-job training for ≥ 25% of ordinary working hours. The eligibility criterion for managers was having had managerial responsibility for the RTW process of one employee included in PROSA.

A strategic sampling of employees of different gender, ages, educational backgrounds, and work sectors was selected from the cohort included in PROSA [39]. During recruitment, the first author contacted 25 employees by telephone. Of these 25 individuals, five did not answer, one did not meet the eligibility criteria, one declined participation, and one cancelled the interview. Employees interested in participating in the study were provided oral and written information during the telephone contact. They were then asked if they agreed to have their manager contacted for participation in the study. If an employee consented, that employee asked their manager and provided the first author with contact details.

Eight employees declined manager participation, citing privacy concerns, workplace changes, or non-work-related sick leave. Therefore, the first author contacted nine managers, all included in employee-manager pairs. Information and consent followed the procedure described for employees. To recruit additional managers and gain a broader understanding of the managerial experience, three additional managers were contacted with the consent of employees included in PROSA. One of these managers was found to match the eligibility criteria and agreed to participate in the present study. Of the resulting total of ten managers, eight were first-line managers, and two were chief executive officers. Despite their various roles, all had been responsible for the RTW process of one employee on sick leave due to CMD. The demographic characteristics of the employees and managers are presented in Table 1.

**Table 1:** Participants’ demographic characteristics

	Employees		Managers	
	Men	Women	Men	Women
Number	8	9	4	6
Age, years, mean (range)	46 (24–55)	44 (34–54)	49 (36–63)	45 (32–54)
Level of education^1^				
Primary/secondary education	5	4	1	0
Higher education/university	3	5	1	6
Work sector				
Private sector	7	4	4	3
Municipality or regional sector	1	5	0	3
Work experience				
≤ 2	4	4	1	0
3–10	2	2	2	4
> 10	2	3	1	2
Living with a partner, *yes*	7	7	NA	NA
Children living at home, *yes*	4	7	NA	NA
Occupational status, *permanent employment*	6	8	NA	NA
Current sick leave, *yes*	2	0	NA	NA
Diagnoses for sick leave, stress/ depression/ anxiety & depression^2^			NA	NA
Adjustment and stress-related disorders (F 43)	4	7		
Depressive disorder (F 32, F 33)	3	2		
Anxiety disorders (F 41)	1	0		
Changes at work after RTW			NA	NA
Part-time work	0	3		
Change of occupation (employment or education)	1	1		

NA = Not applicable

^1^ Missing data for two managers

^2^ Main diagnosis as the reason for sick leave, collected from the Swedish Social Insurance Agency’s register Micro Data for the Analysis of Social Insurance register (MiDAS)

### Data collection

Data were collected by the first author (PhD). She has extensive experience in conducting qualitative interviews. Each participant was interviewed individually on one occasion between October and December 2020. Interviews followed a semi-structured guide and were conducted by telephone. Due to the Covid-19 pandemic, in-person interviews were not possible. The participants were asked to choose a convenient time and to be in a place where they could focus on the interview and speak freely. Based on previous literature [16], interview guides were developed for employees and managers. The guides focused on work and private life and included (a) perceived reasons for sick leave and (b) experiences of facilitators of and barriers to RTW. The analysis of reasons for sick leave has been published elsewhere [13]. Sample questions asked of employees are *“If you think about your work (or private life), what makes it possible to work again?”* and “*Is there anything that makes it difficult to work?”* Sample questions asked of managers are *“If you think about what you told me [perceived reasons for the employee’s sick leave], what possibilities are available to facilitate RTW for the employee at your workplace?”*, “*Is there anything at the workplace that makes it difficult for employees to return after sick leave?”* and *“How do you perceive talking with employees about questions involving work and private life?”* Prompts were used to facilitate an in-depth understanding of the participants’ experiences related to the environment. The participants were asked to elaborate on their and others’ expectations and give examples of situations or cases. Interviews were recorded digitally, and field notes were written after each interview.

### Data analysis

Interviews were transcribed verbatim, and identifying information was removed. The transcripts then were analysed thematically according to Braun and Clark’s six-step process [36, 37]. The thematic approach meant that the researchers focused on interpretation and creation based on understanding data as “context-bound, positioned, and situated” [37, p 591]. The software program N-Vivo 11 was used to organise the data.

The analysis started with the first author checking the accuracy of the transcription, becoming familiar with the data, and noting early ideas. The second and last author also read a selection of interview transcripts. Early ideas documented in field notes were reviewed and discussed among co-authors. In the second step, initial codes were assigned to chunks of interesting and relevant data based on the research aim. Examples of codes are employees sharing *Engaging in activities for recovery and restoration* or managers expressing *I’ve got no tools for this* [supporting RTW]. In the third step, codes were reviewed to find common patterns relating to the aim, forming a basis of early themes and sub-themes. In this step, codes were merged, renamed, and discarded in an iterative process between the dataset, codes, and generated themes. In the fourth step, theme development and review, all authors drew upon their knowledge of multifactorial person-environment aspects of the RTW process, i.e., personal experiences positioned in social and organisational environments. Finally, all authors contributed to steps five and six, namely theme refinement and write-up. The first and second authors compared themes and the dataset, and all authors discussed initial manuscript drafts and the coherence and accuracy of themes.

## Results

The analysis identified three themes with subthemes, each including facilitators of and barriers to RTW in work and private life. The employees’ and managers’ experiences of facilitators of and barriers to RTW identified in the themes represent the experiences of individual struggles and the positioning of the RTW process in social and organisational environments. The themes are named neutrally and encompass a dynamic description of personal, social, and organisational circumstances that facilitate or hinder the RTW process (see Table 2 for an overview of themes).

**Table 2:** Overview of facilitators of and barriers to return-to-work in work and private life after sick leave due to common mental disorders (CMDs) from the perspectives of employees and managers

Themes*	Subthemes	Facilitators of RTW after CMDs	Barriers to RTW after CMDs	Level
Getting along: managing personal difficulties in everyday life		• The capability of identifying and using physical, creative^E^ and cognitive^E,M^ strategies for the space of recovery and restoration • Personal support in identifying difficulties and developing functional strategies (from PHC or OHS)^E,M^	• Symptoms of CMDs negatively affecting work functioning^E,M^ • Perceived lack of psychological capability to manage job demands^M^ • Perceived mismatch between an employee’s psychological capabilities and organisational demands^M^	Personal^§^
Belonging: experiencing social connectedness and support in work and private life	Provision of comfort and space in private life	• Emotional support of a partner, family, and friends providing comfort and guidance in RTW^E^ • Instrumental support of a partner or family member creating space for recovery and restoration^E^ • Emotional and instrumental support of a partner or family member enabling focus on RTW^E,M^	• Lack of, or delayed time to, instrumental support from partner or family members for female employees^E^ • Limited social network restricting access of emotional and/or instrumental support in RTW^E,M^ • Destructive relationships in private life^E^	Social^§^
	Sense of inclusion and teamwork in work life	• Co-workers’ emotional support providing a sense of inclusion in the workplace^E^ • Co-workers’ instrumental support in reducing the employee’s workload^E,M^ • Teamwork for mutual loyalty, shared burdens and responsibilitites^E,M^	• Insufficient transparency with co-workers about CMDs, work accommodations and RTW plan^E,M^ • Managing (the guilt or balance of) the transfer of work tasks to co-workers^E,M^	Social^§^
Organisational support: fostering a supportive work environment	Responsive leadership and dialogue to accommodate the employee	• Responsive leadership and dialogue contributing to: • Accommodation or adjustment through reduction, simplification, and individual tailoring^E,M^ • Managers’ professional advice regarding the employees’ work-tasks and routines^E,M^ • Gradual increase in responsibilities and working hours^E,M^	• Top-down, ready-made adjustments presented for the employee in RTW^E^ • Employees loss of sense- and meaning-making in work when implementing extensive adjustments of worker role^E,M^	Organisational
	Organisational culture and infrastructure to accommodate the employee	• Organisational culture and infrastructure facilitating: ○ Balanced job demands and flexibility to create space for recovery and creativity at work^E,M^ ○ Managerial support (OHS and managerial team)^E^ ○ Availability of staff resources^E,M^	Managers’ insufficient tools to support the employees in the RTW process: • Lack of formal education and organisational guidelines in managing RTW process and CMDs^E,M^ • Lack of external support to increase understanding of employees’ work functioning and RTW-process (PHC & SSIA)^E,M^	Organisational

The table illustrates themes, subthemes, facilitators of and barriers to RTW after CMDs from the perspective of employees^E^ and managers^M^. The table also indicates the level linked to each theme, i.e. personal, social, or organisational. The title of each theme indicates aspects facilitating RTW

Abbreviations: RTW = Return-to-work, CMDs = Common mental disorders, OHS = Occupational Health Service, HR = Human Resource, PHC = Primary Health Care, SSIA = Swedish Social Insurance Agency

^**§**^Personal and social level refers to personal factors of, and social support to, the employee from the perspective of employees and managers

### Getting along: managing personal difficulties in everyday life

On a personal level, employees and managers drew attention to the employees’ persistent difficulties and symptoms of CMDs as barriers to RTW. The RTW process was commonly initiated with a gradual increase of working hours and before the employees had completely recovered. The experienced symptoms (such as fatigue, disturbing thoughts, dizziness, and cognitive problems) during RTW affected their work functioning. For example, symptoms affected the ability to perform specific work tasks, keep the expected work pace, and make priorities at work. For some employees, a relapse back into full sick leave had been necessary, while others said that they kept on struggling:

*I need to double and triple-check everything, so I have some control over things. I must always use a notebook. I have a thousand post-it notes everywhere and such things. I really need my memory and feel sharper working on the things I do. But I feel like I’m getting along anyway.* (Male employee, private sector)

For some employees, symptoms of CMDs were always present. Others felt that increased vulnerability due to demanding situations at work or in private life could trigger symptoms. Managers recognised the personal struggles in RTW after sick leave due to CMDs, and the employees’ difficulties and symptoms of CMDs were identified as barriers to RTW. Managers sometimes framed a dual pressure resulting from the employee’s personal needs during RTW and expectations of organisational efficacy and output. This pressure was experienced as something managers needed to actively manage to support the employees. Overall, employees and managers experienced a poor match between personal factors and organisational demands was a barrier to RTW.

In contrast to the barriers on a personal level, the development and implementation of strategies to manage difficulties in everyday life were seen as facilitators of RTW. These strategies, including the employee’s identification and conscious use of functional approaches, meant to ease the demands of everyday life. Identification involved identifying symptoms, situations that triggered those symptoms, and ways of managing these situations. This identification was achieved independently or through support from health care professionals in primary health care (PHC) or occupational health services (OHS), although access to such support varied among employees. Functional approaches such as engaging in physical or creative activities enabled recovery and restoration. Such engagements could offer a break or distraction from disturbing thoughts and demands in everyday life, and were viewed as building blocks in a daily routine, including work. Therefore, fostering and protecting the space for these engagements were essential in RTW:

*For me, it’s enough to put on my training clothes and walk through the gym door. Because a calm sets in. Then I know I have… yes, but an hour and a half, which is just for me. I am in myself. I do what makes me feel good. (…) It’s probably a lot about being allowed to be alone and with myself, in my body, not in my head [laughs]*. (Female employee, private sector)

In addition to physical or creative engagements facilitating RTW, employees illustrated new ways of thinking and acting in work and private life. Identifying cognitive strategies facilitated creating space for recovery and lowered demands in work and private life, such as accepting restrictions on what could be accomplished during a workday, working at a slower pace, taking regular breaks at work, and lowering expectations of parenting and household responsibilities. Female employees particularly said that it was helpful to lower their expectations. Some life changes that facilitated RTW were unintentional, such as reduced workload or increased flexibility due to the Covid-19 pandemic that allowed more work from home. However, most changes were depicted as intentional and incremental, including intentionally developing strategies for a new rhythm in everyday life in which work was possible.

### Belonging: experiencing social connectedness and support in work and private life

On a social level, the employees’ social support, i.e., emotional and instrumental support in private and work life, are illustrated as facilitators of and barriers to RTW in two subthemes: (a) *Provision of comfort and space in RTW in private life* and (b) S*ense of inclusion and teamwork in work life*.

*Provision of comfort and space in private life.* Employees and managers talked about how emotional and instrumental support in private life facilitated RTW because of the resulting comfort and space to engage in work. Employees said that the experience of emotional support embodied normality and affirmation, free from stigmatisation and shame. Employees and managers reported that emotional support in private life helped employees concentrate more effectively on work-related issues, which ultimately could facilitate a quicker RTW. One female manager working in the municipal sector said: “*The employee had many friends around him, meaning the person returned relatively quickly (…) and the person repeated this to me, “I have much support from home, so you know. You don’t have to worry”* [laughs].” Instrumental support gave the necessary space for recovery and restoration through reduced practical and mental domestic responsibilities (planning for children's activities or caring for older relatives) in private life, and enabled the employees to use the limited energy they had at work. Gender differences relating to social support were noticed among employees. Emotional support was typically offered by a partner (male or female), a mother, a sister, or a female friend. A few men reported positive experiences sharing their emotions with a male friend. Regarding instrumental support, men were given more leeway with household responsibilities during RTW, as such tasks were often removed or made optional.

The lack of equal sharing of responsibilities in private life, especially experienced by female employees, was a barrier to creating the recovery space needed to focus on work. Female employees illustrated various incremental processes, often with some friction, in which they had negotiated support in practical and mental domestic management and responsibilities during RTW. One female employee working in the regional sector said: *“No… I’m trying to change, that he should help more at home and so on… But it’s small steps* [laughs].” Steps toward equal sharing were often related to practical domestic responsibilities. In contrast, equal sharing of domestic mental labour was more challenging. Though managers highlighted a lack of emotional support as a barrier, it was a less tangible barrier among employees. Employees with small networks generally did not view this situation as a barrier but as a fact. However, employees with destructive relationships mentioned these as bothersome and as barriers in RTW.

*Sense of inclusion and teamwork in work life.* Emotional support and instrumental support at work were commonly illustrated as a facilitator of RTW. Employees pointed to the feeling of inclusion when returning to work, such as sharing a day-to-day routine with others and sharing a good collegial atmosphere. Inclusion in a collegial group and a work structure provided familiarity and stability, and was therefore essential for the RTW process. Moreover, employees and managers talked about the instrumental support at work offering relief for the employee during RTW:

*They’ve been amazing and supportive… like never a sigh because they had to pick up the phone or anything like that. “Yes, we will sort it out. We’ll write a little note if there’s something, so you can call them when you have the time.” There were never any problems. And this thing, when you feel like you’re putting your work on someone else, it’s pretty tough because you’re used to doing it yourself.* (Female employee, regional sector)

The quote exemplifies how emotional and instrumental co-worker support contributed to reassurance and reduced workload, facilitating the employee’s focus on her tasks and return to work at her pace. Nevertheless, the quote also points to the risk of adding burdens for co-workers. Instrumental support could accompany feelings of guilt from the employee because of the spill-over of job demands on co-workers. Therefore, teamwork was a preferred solution from the perspectives of employees and managers. Mutual loyalty, support, and well-functioning teamwork meant that employees could use their competencies during RTW without having the sole responsibility at work.

Identified social barriers at work included increased job demands for co-workers supporting the employee and lack of transparency in the team regarding CMD. In line with the employees’ experience of added job demands for co-workers, managers were concerned about balancing the workload among team members during RTW:

*My stress and concern [for the employee and other team-members], I make sure to keep it to myself, and I make sure to get others [team-members] to understand what we must do so that it will be… long-term, hopefully, right.* (Female manager, private sector)

The lack of transparency and communication regarding employees’ mental health and RTW plan could result in discrepancies between reality and team expectations. If the employee was reluctant to disclose their problems to co-workers or the manager failed to communicate the adjustments made, it was difficult for fellow employees to follow the RTW plan. As a result, employees needed to manage more responsibilities than initially anticipated or to cope with perceived inadequacy in co-workers’ eyes if adapting to the RTW plan.

### Organisational support: fostering a supportive work environment

On an organisational level, leadership, culture, and infrastructure were framed as facilitators of and barriers to RTW in two subthemes: (a) *Responsive leadership and dialogue to accommodate the employee* and (b) *Organisational culture and infrastructure to accommodate the employee*. Good working conditions were experienced as creating opportunities for RTW.

*Responsive leadership and dialogue to accommodate the employee*. Responsive leadership enabled a continuous dialogue between the manager and the employee and adjustments for the employee, which facilitated the RTW process. Managers and employees emphasised that a trusting relationship and a transparent dialogue contributed to a mutual understanding of the situation. A transparent dialogue facilitated identification of the employee’s needs in the RTW process and possible solutions for returning to work. As a result of this dialogue, adjustments were designed with a gradual increase in work hours:

*We started very thoughtfully. [The employee] was given limited and isolated tasks for a gentle return. And we had a close dialogue about how it went and felt. Was it too much? Was it too little? How do we allocate working time? When it suits him best? So, generally, we were very flexible and receptive. And, I think, that was also one of the reasons why it went as well as it did.* (Male manager, private sector)

Because high psychological job demands were one reason for employees’ sick leave, adjustments to reduce psychological job demands were important facilitators for RTW. Such adjustments included reducing the number of work tasks, responsibilities, and work pace. Work tasks were often simplified because of employees’ cognitive difficulties and sensitivity to stress. Moreover, employees were given individually-tailored schedules, deadlines, routines, professional roles, and adjustments were made to the physical work environment. Professional advice from the manager about prioritising and redistributing work tasks was also helpful during RTW when the employee had cognitive difficulties and complex work assignments.

On an organisational level, barriers to RTW included top-down, one-size-fits-all solutions and a lack of sense- and meaning-making, as a result of extensive adjustments to accommodate the employee. Employees listed examples of ready-made adjustments that were presented to them without considering specific work responsibilities or their overall life circumstances:

*’Now we have a solution for this’, like… ‘Come along, and we’ll tell you about the solution’. It’s more difficult when they have already figured something out, and then they think, this will be great… Instead of, ‘how do you want to solve this?’* (Male employee, municipal sector).

Because the employees were experts on their situations, poor dialogue with managers could lead to adjustments being perceived of as less feasible or sustainable for the employee. Yet finding the ‘just right balance’ when accommodating the employee was challenging. Adjusting work assignments could involve reduced responsibilities for an employee and stepping down from their previous position, for example, as a project leader. Therefore, it was felt important to consider the impact of such adjustments on the employee’s sense of meaning, worker identity, and career opportunities.

*Organisational culture and infrastructure to accommodate the employee.* The organisational culture and infrastructure were identified as providing potential for accommodating the employee in the RTW process by both managers and employees, and these two factors had the potential to either facilitate or hinder the RTW process. Facilitators were a culture of promoting balanced job demands and the presence of good infrastructure to support the manager and the employee. For example, some managers and employees discussed the organisations’ conscious effort to offset traditional ‘worker ideals’ by avoiding overtime, encouraging recovery, and allowing flexible working hours. Such a culture helped the employee draw boundaries in their RTW process and adapt it to the pace of their recovery and family life. Managers emphasised that balanced work demands created space *during* the workday, enabling recovery, creativity, and innovation. One straightforward solution for facilitating balanced job demands was the availability of staff resources in gradual RTW:

*We must be very clear about what you need to achieve balanced work demands. And if it is the case that you have too much, the idea is that you should get more resources to the extent… that you can complete the task.* (Male employee, private sector)

Managers also named good organisational infrastructure as a facilitator for RTW. Implementing well-functioning routines for managing the RTW process provided managers comfort and guidance in supporting the employee. For example, a human resource (HR) representative could guide a manager through practices and regulations for RTW, or act as a mediator in conflicts. An OHS consult could offer timely counselling to the employee, and both OHS and HR could facilitate the RTW process by participating in the employee-manager dialogue about the RTW. Moreover, a supportive management team functioned as a sounding board for managers. Overall, organisational infrastructure could provide comfort and reassurance that managers were properly supporting the employee in RTW.

Barriers to RTW on an organisational level included insufficient infrastructure or unfavourable working conditions, both of which were linked to the absence of power of the employee and manager. If adverse conditions causing sick leave were left unresolved, employees found themselves in a similar situation during RTW as they did before their sick leave. One employee working in the private sector said: *“I don’t think it’s me who needs medicine; it’s my employer who needs treatment to understand that you can’t have a situation like this at work.”* (Male employee, private sector).

The availability of well-functioning documented routines for RTW, support from HR and OHS consultants, and additional economic resources during RTW varied greatly, and managers specifically mentioned these factors. Some managers felt discouraged by their organisation or their management team. Few managers experienced support from PHC in understanding the specific needs due to CMDs. Most managers said that they needed to know more about CMDs and receive formal training to manage the RTW process. If unsupported, managers had to rely on their intuition and previous experiences with RTWs, and were left with questions of how to support the employee best. One female manager working in the private sector said: “*Did I get it right? Did I get it wrong? How does it work [the RTW-process]? Because I did not experience any support from my immediate superior, instead, as a manager, I was left alone in this.*” (Female manager, private sector).

An employee’s RTW process happened alongside the manager’s other duties, and the added responsibilities sometimes felt difficult and draining for the managers. Lack of support could leave them feeling lonely or constrained. The lack of systematised work environment management and a manager’s lack of power could also be demoralising for the employees because it could delay or hinder resources needed for adjustments. The need for organisational infrastructure supporting the manager was especially highlighted by managers inexperienced in RTW. Managers said that internal and external support helped build a knowledge-based and personalised toolbox to support their employees.

## Discussion

This study identified facilitators of and barriers to RTW in work and private life contexts from the perspectives of employees and managers. Our findings demonstrate that employees struggle with persistent difficulties and symptoms of CMDs during their RTW, and that possible factors affecting RTW span from an individual’s use of strategies in everyday life to an organisation’s culture and infrastructure. The results also demonstrate the situated nature of RTW, i.e., how perceived conditions for RTW are embedded and negotiated within social and organisational structures [40, 41]. Acknowledging the situated nature of RTW is critical because categorising aspects of human experience might risk an unfortunate shift to individualising the problem, which could limit the proper understanding of the situation [40]. However, detangling building blocks in work and private life and positioning them in different contexts might add to our knowledge about the complexity of the RTW process and guide stakeholder actions, including the managers’ and employees’.

The study showed that employees’ persistent CMD symptoms were critical in the gradual RTW process. Persistent symptoms negatively affected work functioning and could be further triggered by high psychological work demands, poor organisational culture, or lack of organisational infrastructure. A negative spiral due to unfavourable person-environment relationships has been reported in research on sustaining work while ill [42, 43] and sick leave due to CMDs [13, 44]. Overall, earlier studies indicate that illness progression, sick leave, and RTW can be seen as a continuum upon which CMD symptoms can accelerate or decrease depending on person-environment relations. This study illustrates strategies for managing symptoms and problematic situations at work and in private life, as opposed to keeping up with demands [13, 43, 44]. These strategies may be used to prevent sick leave or to achieve work sustainability. For example, employees talked about learning to identify triggers of CMD symptoms and implementing creative, physical, and/or cognitive actions necessary for their work functioning. Important aspects of implementing strategies were awareness of problematic situations and being given the space to implement solutions. In line with Danielsson et al. [43], the present study describes an intentional move towards a daily life that incorporates recovery and restoration. Opportunities for implementing changes in everyday life were negotiated and shaped between the person and their environment. For example, healthcare support was imperative for many employees to identify problematic situations and strategies, yet the access to healthcare support varied. Moreover, in line with previous studies [16, 22, 45], opportunities could unfold through support from significant others. However, RTW could also be hindered by traditional gender ideals and divisions of labour. In line with previous reports on women’s experiences [46, 47] and gender differences [16] in the RTW process, women in this study had less leeway in family responsibilities and fewer opportunities for recovery and restoration.

Other critical parts of the RTW process were support and accommodations at the workplace, echoing how reduced working hours alone are insufficient in RTW [26]. The need for individually tailored RTW after sick leave due to CMDs, including work adjustments, is continually reported [16, 22, 26, 30]. We found that RTW facilitators include reduced workload and responsibilities, simplified tasks, and flexible schedules. It has been widely reported that communication is essential for accommodating the employee [22, 23, 27, 28, 30]. In this study, responsive leadership and continued dialogue were found to be central to work functioning. These findings resonate with “compassionate leadership” [30] and expressions of the managers’ recognition of the employee [19], which has been identified as fundamental to a respectful and trusting relationship in negotiating solutions for RTW after CMDs [30], and confirms reports of managerial support as a complex challenge [23, 27]. Additionally, we identified several barriers to the RTW process relating to the managerial role, including pre-made adjustments, too extensive adjustments, and a lack of transparency. These barriers could cause an employee to feel inadequate or as if there was no sense-making in work. Unsatisfactory adjustments or poor transparency could also be a reason for insufficient adherence to the employee’s RTW plan or discrepancies in expectations between the returning employees and their co-workers, as described by Farias et al. [48]. Teamwork was regarded as a solution that could counteract such barriers because of access to various competencies and shared responsibilities in the team. Prior research has shown that the team in RTW can provide a wider supportive environment for the employee [23]. Potentially, teamwork can offset the individualisation of responsibility for RTW, and be essential for a manager in handling the varying pressures relating to employees’ and co-workers’ needs, as reported in this and other studies [27, 29].

The conditions for RTW must be critically reviewed from an organisational perspective [10, 23, 27]. Our results suggest that recognising organisational culture and infrastructure would be helpful for accommodating the employee. To improve conditions for RTW, managers and employees felt that traditional worker ideals (such as the idea that employees should be entirely devoted to their work and unburdened by other obligations) [49] needed to be revised. Rather, employees and some of the managers advocated for a culture that balanced job demands and incorporated flexibility. In addition, a sufficient organisational infrastructure could create conditions for essential employee-manager dialogues. In line with other research [23, 27, 29], our study found insufficient manager training and support in RTW. We suggest that organisational learning, i.e., a process in which knowledge and routines are critically reviewed and gradually embedded in practice, can potentially improve RTW processes [50]. For example, organisational measures such as developing and actively using policies and routines in the RTW process are suggested to be a supportive factor for managers [19, 27]. Organisational learning should also include managerial support and formal manager training. Training could consist of knowledge of risk factors for CMDs, prolonged RTW, and factors associated with RTW [10, 51]. Training could also include communication and problem-solving techniques [51]. Moreover, support from HR, OHS consultants, and a management team is essential for helping to offset manager’s loneliness in RTW. Our results also show that organisations must review manager support in light of line managers’ authority. Lacking the authority to make decisions or support by senior management, managers felt constrained in trying to meet the needs of employees. If the organisational structure is insufficient, the results of this study indicate that there can be health risks for managers in managing RTW due to insecurity, powerlessness, and a high workload.

In summary, as Nielsen et al. [18] theorised, utilising resources at different levels in work and non-work contexts is likely required to improve RTW. Our examples show that healthcare and significant others can give valuable employee support in implementing strategies and enabling room for recovery and restoration. Moreover, healthcare can also support the employee by informing an employee’s manager about CMDs and their individual needs. However, symptom management is not the sole solution for RTW [52]. Positive experiences of working against traditional worker ideals [49], suggest a new approach for both manager and employee– namely, to acknowledge that employees’ everyday lives go beyond work and are, at times, challenging, rewarding, and sources of energy. For example, space during the workday could be helpful for those who reported primarily work-related causes for their sick leave and those burdened by their private life engagements with less instrumental support, as reported here and also by previous research [13, 47].

The primary strength of the present study is the focus on the most proximate stakeholder perspectives on RTW after sick leave due to CMDs. Involving managers and employees representing a variety of ages, educational levels, work sectors, and genders provides a rich data set [53]. It is possible that involving other stakeholders, such as colleagues, family, and friends, would provide an even broader understanding of work and private life during RTW. However, in this study, we chose to limit the data collection to employees and managers. Another strength is its positioning of barriers and facilitators in social and organisational environments. In addition to helping us understand how to support RTW after CMDs, this approach might bridge rehabilitation and prevention approaches [7] and contribute to avenues for work sustainability.

A limitation of the study is that the employees recruited for this study were included in a randomised trial with the eligibility criterion “acceptance of manager involvement” [39]. There is, therefore, a potential risk of response bias due to employees with problematic relationships with their managers declining participation. Another limitation is the risk of recall and social desirability bias because of the retrospective interviews and social norms of work. To counteract those forces and achieve authentic responses, information about confidentiality was repeated often, and the interviewees were encouraged to choose an environment where they could speak openly [54]. Moreover, to elicit rich data, participants were guided to situate their responses in context by examples or cases [53].

The rich data set, thorough engagement with the data during coding and theme development, and discussions among our interdisciplinary team of researchers facilitated thick descriptions and a complex understanding of RTW [53, 55]. The results are likely transferable to other Swedish settings. Although the context of RTW differs among countries, the similarities between our results and earlier research indicate that elements in RTW are transferable across countries. Our detailed descriptions of the study design facilitate the judgement of transferability [53].

## Conclusion

The study explores RTW after CMDs from a multi-stakeholder perspective and from an understanding of the everyday as an arena where RTW occurs. The results contribute to a broad understanding of the RTW process, including the experience of an individual struggling in work and private life contexts. Based on the results, RTW can be seen as an active and ongoing process, where RTW is a collective endeavour embedded in social, organisational, and societal environments. The results contribute to insights about the “work first” mentality in creating strategies for RTW– namely, that the influence of organisational culture and infrastructure must be reviewed in accommodating employees on sick leave due to CMDs. Such strategies include manager support, formal manager training, and providing returning employees space to recover during the workday. Highlighting a “work first” mentality also includes considering internal and external support for managers to fulfil the employer’s responsibilities in RTW. These strategies seem overarching in creating good conditions for RTW.

## Data Availability

The datasets generated and analysed during the current study are not publicly available due to the Swedish ethical review regulation. Data are available upon reasonable request. Inquiries for data access should be sent to Karolinska Institutet, Institute of Environmental Medicine, Unit of Intervention and Implementation Research for Worker Health, Box 210, 171 77 Stockholm or contact the principal investigator Elisabeth Björk Brämberg, elisabeth.bjork.bramberg@ki.se, who will then contact the Swedish Ethical Review Authority for permission.

## Abbreviations

CMDs: Common mental disorders
HR: Human Resources
RTW: Return-to-work
OHS: Occupational Health Services
PHC: Primary Health Care
SSIA: Swedish Social Insurance Agency
